# Supplementary material for: A Pilot Study on the Effects of Transcranial Direct Current Stimulation on Brain Rhythms and Entropy during Self-Paced Finger Movement using the Epoc Helmet
Source: Front Hum Neurosci. 2017 Apr 25;11:201. doi: 10.3389/fnhum.2017.00201 (PMC5408787; doi:10.3389/fnhum.2017.00201)
Supplement: Supplementary file 1 [file Data_Sheet_1.zip › Complementary_results/Kullback-Leibler-Divergence_Rest-Referenced_average_PSD_windows/Kullback-Leibler_Rest-Referenced_avg-win_Avg F4-F8-FC6.pdf]

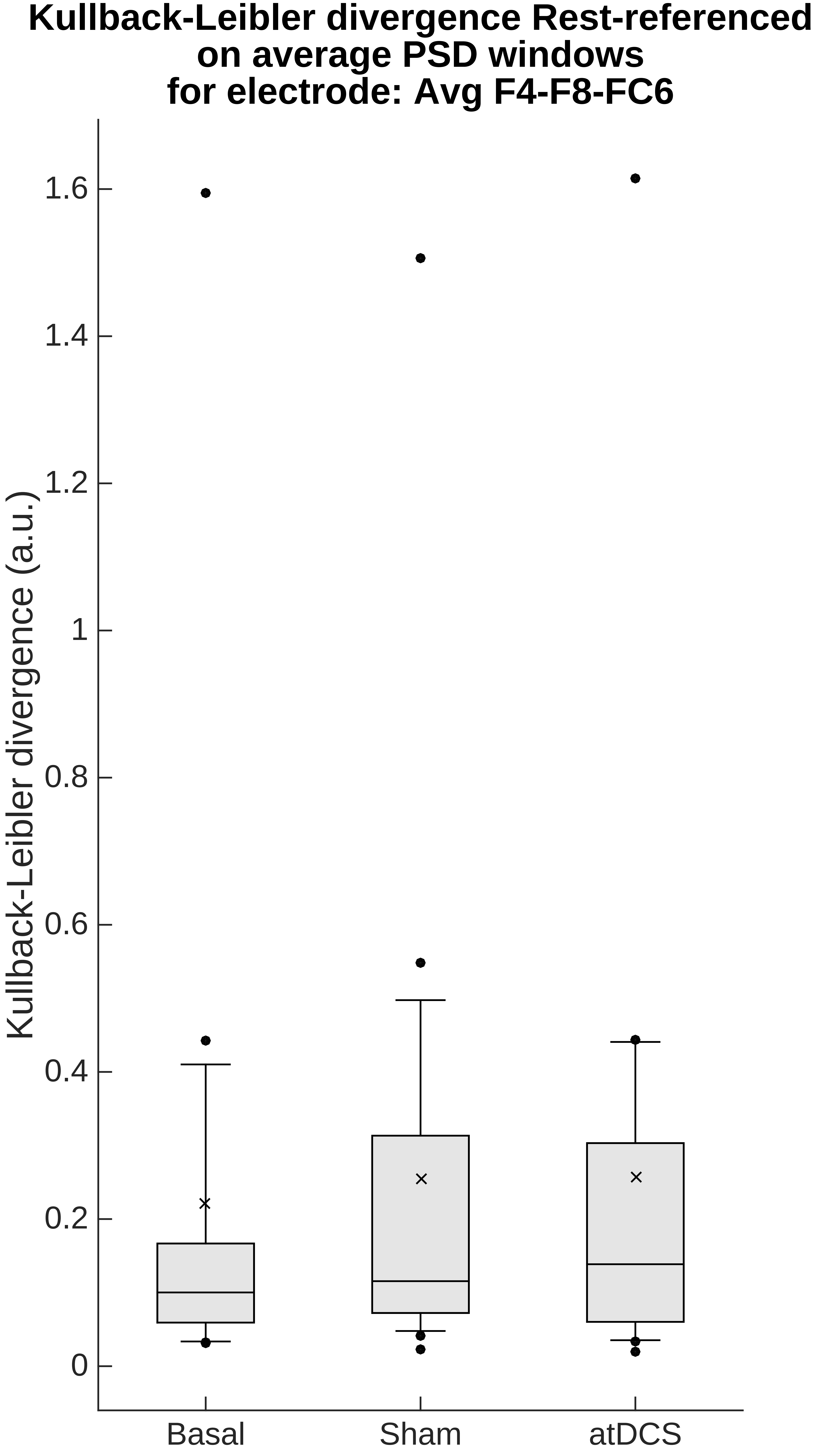
Kullback-Leibler divergence Rest-referenced
on average PSD windows
for electrode: Avg F4-F8-FC6
Kullback-Leibler divergence (a.u.)
0
0.2
0.4
0.6
0.8
1
1.2
1.4
1.6
Basal
Sham
atDCS
